# Immunoevolution of mouse pancreatic organoid isografts from preinvasive to metastatic disease

**DOI:** 10.1038/s41598-019-48663-7

**Published:** 2019-08-22

**Authors:** Dea Filippini, Sabrina D’ Agosto, Pietro Delfino, Michele Simbolo, Geny Piro, Borislav Rusev, Lisa Veghini, Cinzia Cantù, Francesca Lupo, Stefano Ugel, Francesco De Sanctis, Vincenzo Bronte, Michele Milella, Giampaolo Tortora, Aldo Scarpa, Carmine Carbone, Vincenzo Corbo

**Affiliations:** 1 Department of Diagnostic and Public Health, University of Verona, Verona, Italy; 2 ARC-Net Research Centre, University of Verona, Verona, Italy; 3 Department of Medicine, Section of Medical Oncology, University of Verona, Verona, Italy; 4 Department of Medicine, Section of Immunology, University of Verona, Verona, Italy; 5 Present Address: Fondazione Policlinico Universitario A. Gemelli IRCCS, Rome, Italy; Università Cattolica Del Sacro Cuore, Rome, Italy

**Keywords:** Cancer models, Cancer

## Abstract

Pancreatic ductal adenocarcinoma (PDA) has a highly immunosuppressive microenvironment, which is contributed by the complex interaction between cancer cells and a heterogeneous population of stromal cells. Therefore, facile and trackable models are needed for integrative and dynamic interrogation of cancer-stroma interaction. Here, we tracked the immunoevolution of PDA in a genetically-defined transplantable model of mouse pancreatic tumour organoids that recapitulates the progression of the disease from early preinvasive lesions to metastatic carcinomas. We demonstrated that organoid-derived isografts (ODI) can be used as a biological source of biomarkers (*NT5E*, *TGFB1*, *FN1*, and *ITGA5*) of aggressive molecular subtypes of human PDA. In ODI, infiltration from leukocytes is an early event during progression of the disease as observed for autochthonous models. Neoplastic progression was associated to accumulation of Maf^+^ macrophages, which inversely correlated with CD8^+^ T cells infiltration. Consistently, levels of *MAF* were enriched in human PDA subtypes characterized by abundance of macrophage-related transcripts and indicated poor patients’ survival. Density of MAF^+^ macrophages was higher in human PDA tissues compared to preinvasive lesions. Our results suggest that ODIs represent a suitable system for genotypic-immunophenotypic studies and support the hypothesis of MAF^+^ macrophages as a prominent immunosuppressive population in PDA.

## Introduction

Pancreatic ductal adenocarcinoma (PDA) is a deadly disease with a 5-year survival rate less than 6% due to late diagnosis and poor responsiveness to available therapies^1^. Although being the tenth in incidence, PDA is the fourth leading cause of cancer death, and it is estimated to become the 2nd cause of cancer deaths within a decade^2^. Differently from other solid tumours^3,4^, immunotherapeutic strategies have proven ineffective in the majority PDA, with the exception being patients with high microsatellite instability^5^. Many factors contribute to the creation of a highly immunosuppressive microenvironment that limit cancer immunotherapy efficacy (e.g., immune-checkpoint inhibitors), including poor tumours immunogenicity, a heterogeneous and complex tumour microenvironment, and plasticity of PDA cells.

A recent transcriptomic analysis of primary PDA has identified 4 molecular subtypes (ADEX, Pancreatic Progenitor, Squamous, and Immunogenic)^6^, which expanded the previous two-group classification (Basal-like and Classical) proposed by Moffit *et al*.^7^. Based on intrinsic characteristics of neoplastic cells, PDA can be reliably classified as Squamous/Basal-like or Classical/Pancreatic Progenitor, whereas ADEX and Immunogenic subtype appear to be driven by transcripts from non-neoplastic cells^8^. Moreover, these tumour subtypes show different prognosis and different response to therapy^7,9^. Of the four subtypes, two are enriched for immune-related signatures: Immunogenic and Squamous^6^. The Squamous subtype, which is enriched for squamous and poorly differentiated cancers, carries the poorest prognosis and shows signatures indicative of an immune suppressive microenvironment, including elevated macrophage gene expression programs^6^. Recent studies have shown that it is possible to induce a “class switch” from Squamous to more differentiated and more immunogenic subtypes by targeting either cancer cell features^10^ or selected immune populations^11,12^. It is also becoming clear that cancer cell programs driven by specific genetic events are able to dictate the immune landscape of cancers^13^, including PDA^14^. Therefore, an integrated analysis of genetic features and immunophenotypes is required to identify undisclosed immunotherapeutic opportunity in PDA. This possibility has been limited, at least in part, by the difficulty in creating complex preclinical models for easy and rapid testing of genotype-driven immunological changes. Genetically engineered mouse models (GEMMs) of PDA have demonstrated to faithfully recapitulate major pathophysiological features of the human disease, including the prominent stromal reaction that also involves immune cells^15,16^. An alternative approach might be to introduce specific genetic alteration *ex vivo* and then exploit syngeneic cell transplants. However, preclinical mouse models generated through implantation of syngeneic monolayer cell cultures cannot be used to track the changes in the immune microenvironment during progression from early preinvasive lesions to invasive and metastatic carcinomas. Moreover, cell line-based transplants often fail to produce the fibroinflammatory reaction that characterize the majority of PDA^17,18^. Recently, it has been shown that orthotopic transplants of pancreatic tumour organoid slowly progress from preinvasive lesions to invasive carcinoma^17^. Similar to GEMM, organoid derived isografts recapitulate some of the relevant features of human PDA including vascularization and stromal deposition^17^. Nonetheless, whether and how disease progression in organoid-derived isografts (ODI) is associated to accumulation of genetic alterations as well as to changes in the immune contexture is currently unknown, thus limiting the usefulness of this system for translational studies. Here, we characterized the cellular components and the dynamic changes of the immune contexture during progression of pancreatic tumour organoid derived isografts.

## Results

### Histopathological evolution of organoid derived isografts

Using established procedures^17,19^, we generated organoid cultures from the pancreata of wild-type C57Bl/6 mice (n = 3) and from pancreatic tissues of KPC (Kras^+/LSL-G12D^; Trp53^+/LSL-R172H^; Pdx1-Cre)^16^ mice (n = 3), which contained invasive PDA (Fig. S1a). Although indistinguishable at microscopic evaluation (Fig. S1b), normal- and tumour-derived cultures were genetically different. Targeting sequencing of PDA driver genes (19 genes, Table S1) identified mutations of *Kras* and *Trp53* in tumour derived organoids (B6-Ks), thereby confirming that these two events are sufficient for the development of PDA in mice and are maintained in culture^16^. As expected, normal organoid cultures (B6-Ns) were devoid of mutations in the cancer genes analysed (Fig. S1c).

It has been previously demonstrated that orthotopic transplants of mouse tumour organoids in syngeneic immunocompetent mice slowly progress from preinvasive lesions (PanIN-like lesions) to invasive carcinomas^17^. Nonetheless, whether and how disease progression in organoid-derived isografts (ODIs) is associated to accumulation of genetic alterations as well as to changes in the immune contexture is currently unknown.

**Figure 1:**
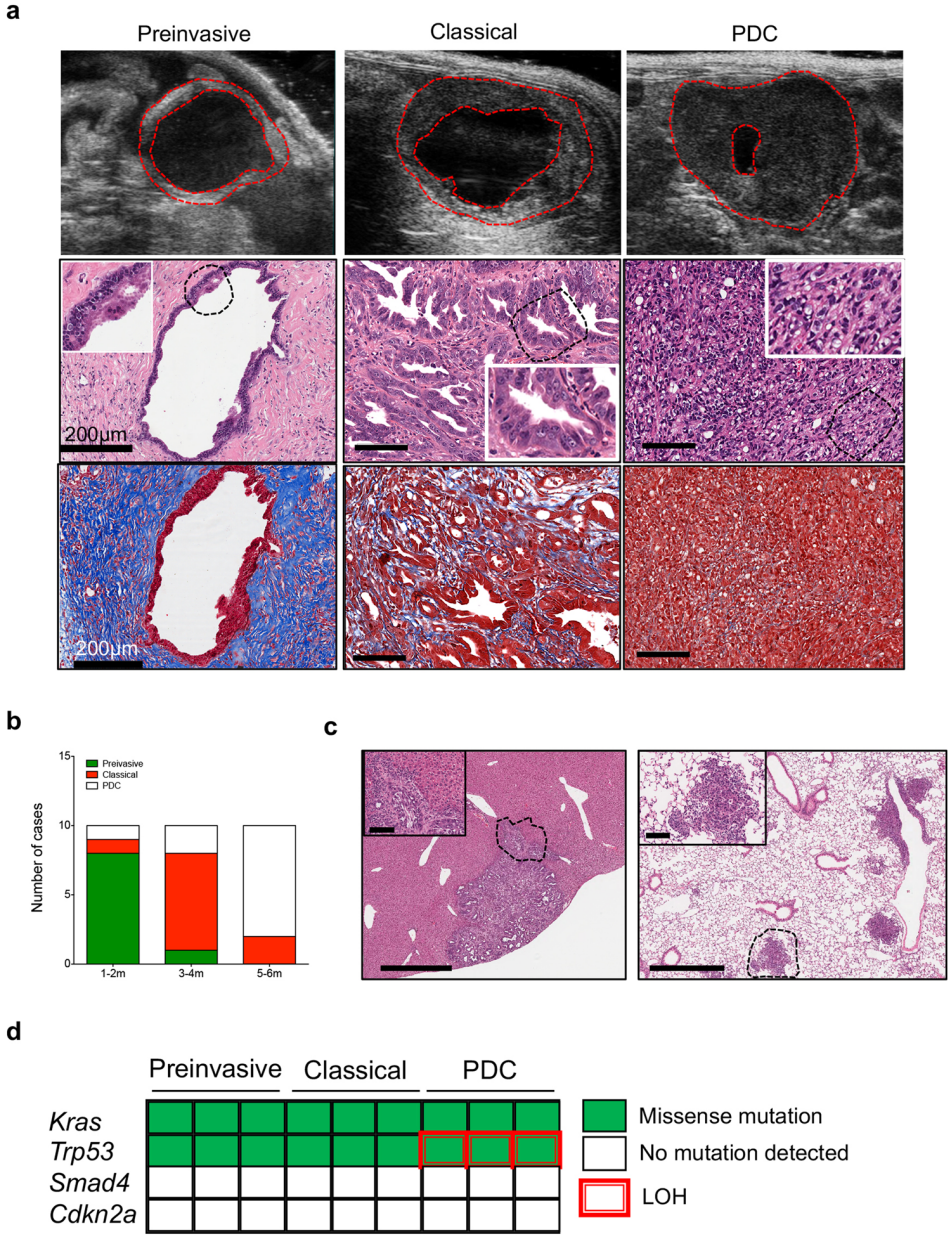
Histopathological evolution of tumour organoid isografts. (**a**) The growth of tumour organoid isografts was monitored by high-contrast ultrasonography (top); tumours are outlined in red. Representative hematoxylin & eosin staining (middle) of tissues from a preinvasive lesion, a moderately differentiated tumour (classical), and a poorly differentiated carcinoma (PDC) obtained at 1, 3, and 5 months, respectively, from orthotopic transplantation of B6-K1 mouse organoids. Scale bars, 100 µm unless otherwise indicated. Magnification of selected areas (dashed lines) is provided in the insets. Preinvasive lesions presented an intense desmoplastic reaction compared to moderately and poorly differentiated tumours by Masson’s trichrome staining (bottom). Scale bars, 100 µm unless otherwise indicated. **(b)** Bar graph showing the number and type of lesions observed in the pancreas of immunocompetent mice at different times from transplantation (m, months). **(c)** Representative metastatic growths at the liver (left) and at the lungs (right) from a mouse bearing a poorly differentiated carcinoma (B6-K2 organoid, 3 months from transplantation). The dashed lines indicate the areas shown in the insets. Scale bar, 700 µm; insets, 100 µm. **(d)** Targeted sequencing of preinvasive (n = 3), classical tumours (n = 3), and poorly differentiated carcinomas (n = 3) derived from mouse B6-Ks orthotopic transplants. The status of the 4 more commonly mutated PDA genes is shown with colour key providing information on type of alteration. LOH, loss of heterozygosity. See also Supplementary Fig. S1.

To evaluate the histopathological progression of organoid derived isografts, we generated an array of 30 ODIs from 3 individual organoid cultures (Fig. S1d). The growth of ODIs was monitored using high-contrast ultrasonography, which revealed that organoid transplants initially grew as small solid lesions developing from a cystic structure, recognizable as spherical black regions with distinct borders (Fig. 1a). Over long period of time (from 1 to 6 months), the cyst eventually reduced in size. First, we analysed the histological features of tumours from ODI at early (1–2 months), intermediate (3–4 months), or late (5–6 months) time points following transplantation. Initially, the majority of ODIs developed low- to high-grade preinvasive lesions that cytoarchitecturally resembled mouse PanIN including an abundant deposition of stroma. Over time (3 to 4 months post transplantation), ODIs progressed into “classical” mouse PDA, which encompassed well- and moderately differentiated tumours with prominent stromal deposition. At later time points (5 to 6 months post transplantation), ODIs were mostly poorly differentiated carcinomas (PDC) containing areas with sarcomatoid features and almost no stroma deposition (Figs 1a,b, S1e,f). At any of the time points considered, PDC were the only ODI to present with metastases at the liver and the lungs (Fig. 1c). To ask whether this histological progression was associated to accumulation of alterations in PDA genes, we applied targeted sequencing of 19 genes to lesions from ODI and found that, except for loss of the wild-type copy of *Trp53* in PDC, no other genetic alteration accumulated *in vivo* (Fig. 1d). Loss of heterozygosity of *Trp53* in metastatic PDC is consistent with previous observations made in the KPC model^16^.

### Immunological evolution of organoid derived isografts

**Figure 2:**
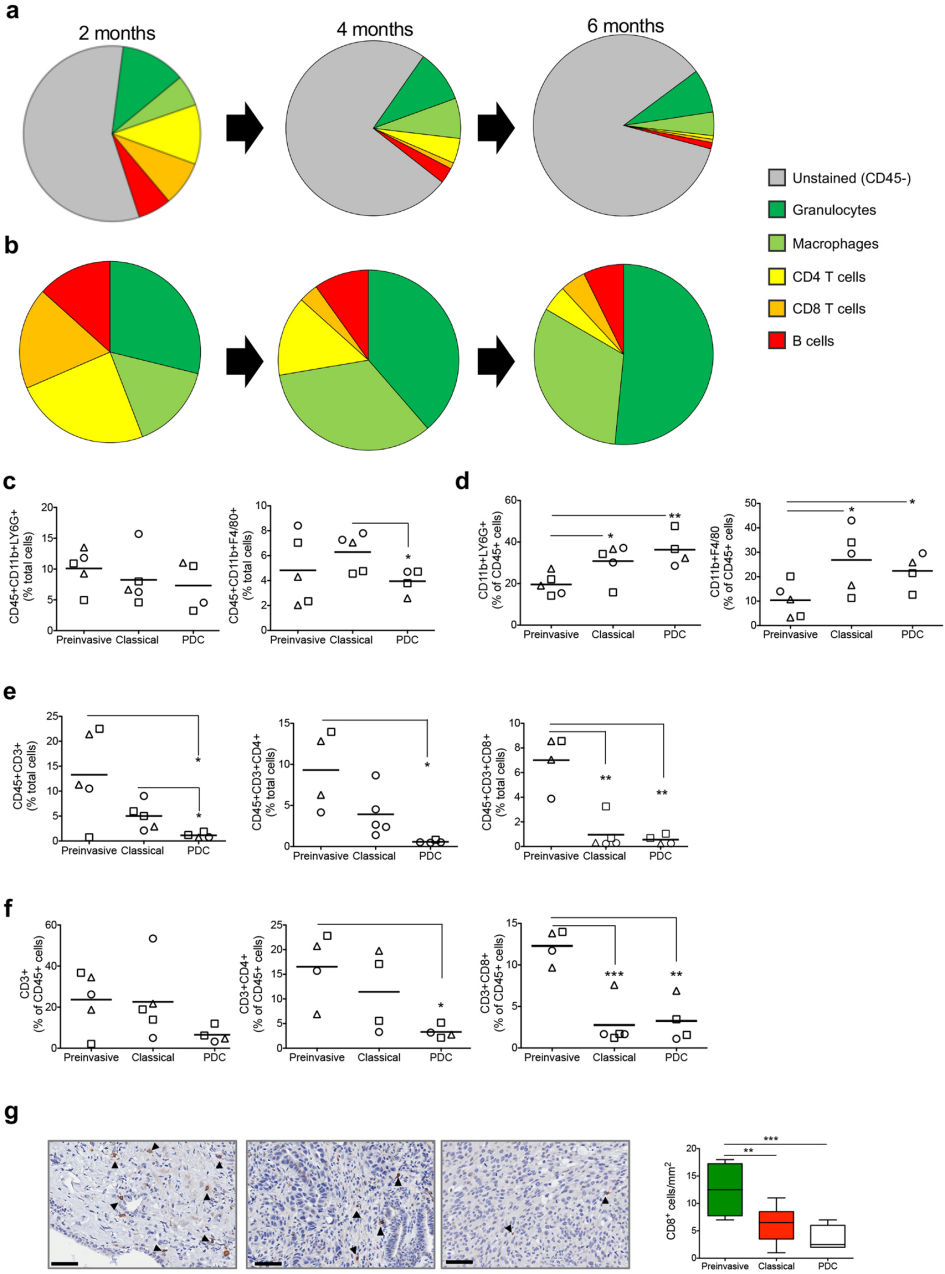
The composition of immune infiltrates in tumour organoid derived isografts. (**a**) Pie charts showing percentages of granulocytes (CD45^+^CD11b^+^Ly6G^+^), macrophages (CD45^+^CD11b^+^F4/80^+^), B cells (CD45^+^B220^+^), cytotoxic T cells (CD45^+^CD3^+^CD8^+^), and T helper lymphocytes (CD45^+^CD3^+^CD4^+^) in the pancreatic tissues from mice at 2 months (left), 4 months (middle) and 6 months (right) from organoid transplantation. “Unstained” denotes pancreatic epithelial cells and other stromal components. Colour key is provided. **(b)** Pie charts showing the immune cell populations defined in (**a**) as percentages of CD45^+^ cells. Colour key as in (**a**). **(c)** Flow cytometry analysis of the indicated CD45^+^ cell populations (granulocytes and macrophages) in the pancreas of preinvasive, classical tumours, or PDC from (**a**). **(d)** Flow cytometry analysis of the indicated immune cell populations (granulocytes and macrophages) in the pancreas of preinvasive, classical tumours, or PDC from (**b**). **(e)** Flow cytometry analysis of the indicated CD45^+^ cell populations (T cells) in the pancreas of preinvasive, classical tumours, or PDC from (**a**). **(f)** Flow cytometry analysis of the indicated immune cell populations (T cells) in the pancreas of preinvasive, classical tumours, or PDC from (**b**). Each dot in the graphics (**c**–**f**) refers to the individual tumour samples available for cytometric evaluation. ODIs from B6-K1, B6-K2, and B6-K3 organoids are identified by circles, triangles, and squares, respectively. (**g**) Immunohistochemical staining for CD8 in tissue from mice bearing preinvasive lesions (B6-K2, left), classical tumours (B6-K2, middle) or poorly differentiated tumours (B6-K3, right). Scale bars, 50 µm. Quantification is provided on the left as the average number of CD8 positive cells per mm^2^ in preinvasive (n = 6), classical (n = 10), and poorly differentiated (PDC, n = 8) tumours. From 3 to 5 individual areas per case were examined. Statistical associations were determined by Student’s t-test. *p < 0.05; **p < 0.01; ***p < 0.001. See also Supplementary Fig. 2 for details.

To address whether progression of ODI was associated to changes in the immune landscape, we applied a multidimensional tumour profiling approach to whole pancreatic lesions from ODI sacrificed at different times post transplantation as previously indicated. First, we analysed the composition of immune populations by cytofluorimetric analysis. We found substantial changes in the total number (Fig. 2a) and relative composition (Fig. 2b) of leukocytes (CD45^+^ cells) in pancreatic tissues from mice bearing lesions at different stages of disease. In the pancreata containing preinvasive lesions and classical tumours, CD45^+^ cells represented the 51.7 ± 13.06% and 37.2 ± 9.24% of total cells, respectively, whereas a substantial decrease in total leukocytes was observed in PDC (19.50 ± 8.6%) (Fig. S2a). As previously shown in KPC mice^15^, the increased infiltration of leukocytes compared to normal pancreas was associated to the reduction of CD45 expressing cells in the spleen of mice bearing pancreatic lesions compared to lesion-free mice suggesting an enforced recruitment of leukocytes from lymphoid tissue to the tumour mass (Fig. S2b).

**Figure 3:**
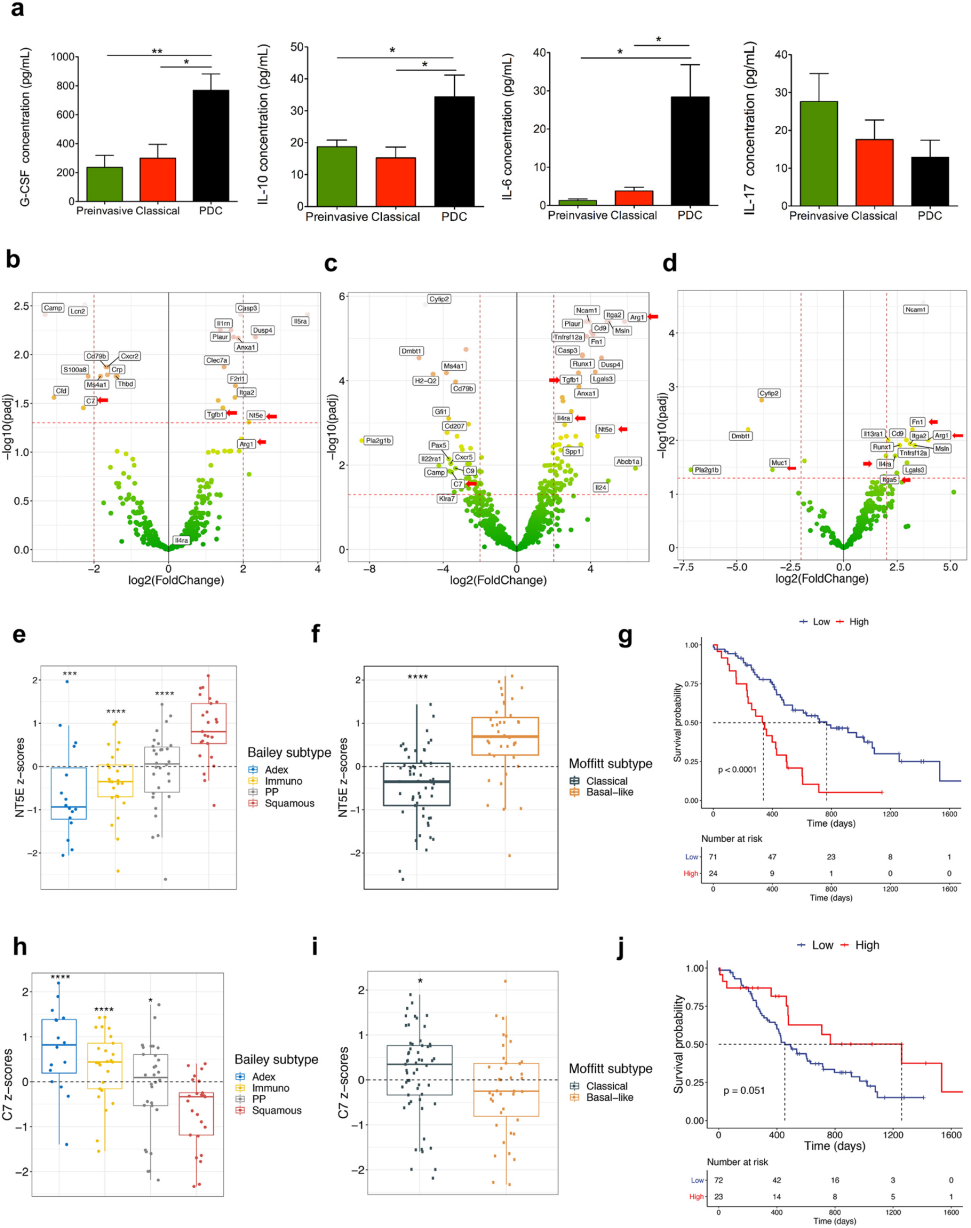
Disease progression of organoid-derived isografts is associated with gene expression changes indicative of an immunosuppressive microenvironment. (**a**) Multiplex bead-based mouse cytokine assay for serum detection of circulating factors in mice bearing preinvasive lesions (n = 5), classical tumours (n = 6) and poorly differentiated tumours (PDC, n = 5). Mean and SEM in pg/mL are shown. See also Supplementary Fig. 3. **(b)** Volcano plots of differences in gene expression (NanoString platform) between classical tumours (n = 3) and preinvasive lesions (n = 3). Indicated are the genes with Log2 fold change in expression ≥ 1.5 and adjusted p < 0.05. (**c**) Volcano plots of differences in gene expression (NanoString platform) between poorly differentiated tumours (n = 3) and preinvasive lesions (n = 3). Indicated are the genes with Log2 fold change in expression ≥ 2 and adjusted p < 0.05. (**d**) Volcano plots of differences in gene expression (NanoString platform) between poorly differentiated (n = 3) and classical (n = 3) tumours. Indicated are the genes with Log2 fold change in expression ≥ 2 and adjusted p < 0.05. In (**b**,**c**, and **d**), red arrows indicate genes that are discussed in the text. (**e**,**f**) Box plot showing the *NT5E* Z-score score stratified by Bailey (**e**) or Moffitt subtypes (**f**) in the ICGC cohort. ****p ≪ 0.001; ***p < 0.001; *p < 0.05 as determined by Wilcoxon rank-sum test. (**g**) Kaplan–Meier analysis comparing survival of patients in the ICGC cohort having either high or low expression of *NT5E*. p, Log-rank (Mantel-Cox) test. (**h**,**i**) Box plot showing the *C7* Z-score score stratified by Bailey (**h**) or Moffitt subtypes (**i**) in the ICGC-PDA cohort. ****p ≪ 0.001; *p < 0.01 as determined by Wilcoxon rank-sum test. (**j**) Kaplan–Meier analysis comparing survival of patients in the ICGC cohort having either high or low expression of *C7*. p, Log-rank (Mantel-Cox) test. See also Supplementary Figs 5 and 6.

Using established markers of immune cell populations, we found that granulocytes (CD11b^+^Ly6G^+^) and macrophages (CD11b^+^F4/80^+^cells) heavily infiltrated preinvasive lesions and persisted throughout tumour progression (Figs 2c and S2c). When looking at the relative composition of the CD45^+^ cells infiltrating ODIs, the myeloid populations significantly expanded during progression (Figs 2d and S2d). High density of lymphocytes, in particular of CD8^+^ T cells, has been associated to long-term PDA survivors^20^. Therefore, we sought to assess the degree of infiltrating lymphocytes in our ODI and found a dramatic reduction of T lymphocytes (CD3^+^cells), T_H_ CD4^+^ T cells, and particularly of cytotoxic CD8^+^ T cells, in tumours compared to preinvasive lesions (Fig. 2e). In keeping with this, CD8^+^ T cells were the lymphocytic population to more dramatically reduce during progression, both in classical tumours and PDC, when looking at relative composition of CD45^+^ infiltrating cells (Fig. 2f). These results were corroborated by immunohistochemistry, which showed a significant reduction in the number of CD8^+^ T cells in tumours compared to preinvasive lesions (Fig. 2g). Prominent B lymphocytes infiltration has been previously described to occur early during pancreatic neoplasia and to exert a pro-tumorigenic role^21^. In keeping with this, we found that B lymphocytes (B220^+^ cells) were detectable in both early lesions and classical tumours while decreasing substantially in PDC (Fig. S2e,f). The T helper subset was the most abundant lymphocytic population in preinvasive lesions (Fig. 2a), which prompted us to investigate whether it contained immunosuppressive T_reg_ cells^22^. We used immunofluorescence to co-localize T_reg_ (Foxp3) and cytotoxic T cells (CD8) in tissues from different stages of disease and found that T_reg_ cells infiltrate early, persist throughout tumour development, and outnumber CD8^+^ T cells in each stage of ODI (Fig. S2g,h,i). Overall, these data suggest that myeloid cells infiltrate early and persist throughout ODI progression, while T cells are excluded as tumour progresses. These changes in immune infiltration of the tumour were associated to specific changes in circulating cytokines/chemokines, with the level of G-CSF and anti-inflammatory cytokines (IL-6 and IL-10) increasing in PDA compared to preinvasive lesions (Figs 3a and S3). Interestingly, we also found higher circulating levels of IL-17 in preinvasive lesion compared to tumours (Fig. 3a). Previous studies have demonstrated that IL-17 produced by infiltrating immune cells is necessary for initiation and progression of PanIN^23,24^.

### Immunosuppression increases as tumours progress in the ODI system

T cell exclusion and T cell disfunction are two major mechanisms through which tumours are able to escape immune control^25–27^. Recent works have highlighted the possibility of using mRNA-based signatures as biomarkers indicative of T cell exclusion/disfunction^28,29^. Therefore, we performed targeted gene expression profiling, using a commercially available NanoString panel composed of immune-related genes, in FFPE samples from preinvasive, classical and PDC ODIs. When comparing expression profiles (EP) from preinvasive lesions (n = 3) and classical tumours (n = 3), 24 genes were found deregulated (adjusted p < 0.05, Supplementary Table 2). Among most upregulated genes in classical tumours, *Nt5e*, *Tgfb1*, and *Arg1* (Figs 3b and S4a) have been consistently associated to T cell disfunction^29–33^. In particular, *Nt5e* (also known as CD73) is expressed by cancer cells and encodes for an ectonucleotidase that cooperates with CD39 to generate extracellular adenosine, which can in turns prevent T cells activation and proliferation^34^. Differently from *Nt5e*, *Arg1* is expressed by immunosuppressive myeloid cells and encodes a critical enzyme which converts L-arginine to urea and Lornithine. L-arginine consumption blocks the lymphocyte cell cycle and at the same time it promotes the blockade of the ζ chain of CD3, which prevents T cells from responding to various stimuli^33^. Among downregulated genes in classical tumours compared to preinvasive lesions (Figs 3b and S4a), *C7* encodes for a component of the complement cascade and its reduced expression has been reported as a poor prognostic marker in several malignancies^35,36^.

When comparing EP from PDC and preinvasive lesions, 109 genes were found deregulated (adjusted p < 0.05, Supplementary Table 2). Upregulation of *Nt5e*, *Tgfb1*, *Arg1* and downregulation of *C7* was also observed in this comparison (Figs 3c and S4b), along with increased expression of *Il4ra* (Figs 3c and S4b), which encodes for the alpha chain of the Interleukin 4 receptor and it is a well-established marker of immunosuppressive myeloid cells^37^. Fewer genes were found deregulated when comparing EP from PDC and classical (Figs 3d and S4c), and *Arg1* and *Il4ra* were among the genes further upregulated suggesting that immunosuppressive myeloid cells were accumulating as tumour progressed. In addition, *Muc1* was downregulated, while *Fn1* and *Itga5* were upregulated in PDC compared to classical tumours (Fig. 3d). Details about shared and unique genes up- or down-regulated comparing EP from ODI at different disease stages is provided in Fig. S4e and Supplementary Table 3. Deregulation of selected genes (*Arg1*, *C7*, *Lcn2*, *Nt5e*, and *Tgfb1*) from preinvasive lesion to overt cancers (classical tumours and PDC) was orthogonally confirmed by qPCR (Fig. S4f).

It should be noted that while increased expression of *Nt5e* and *Tgfb1* could be ascribed to the increase in the neoplastic cells content from preinvasive lesions to PDC, increased expression of immune cell genes *Arg1* and *Il4ra* clearly suggest differentiation of pre-existing myeloid cells into immunosuppressive phenotypes during ODI progression.

### ODI represents a valid biological resource for identification of clinically-relevant biomarkers

To assess whether interrogation of EP of ODIs at different disease stages might help identifying genes relevant to the human disease, we interrogated the transcriptomes of PDA from the International Cancer Genome Consortium (ICGC) and The Cancer Genome Atlas (TCGA) for differential expression of selected genes (*C7*, *MUC1*, *NT5E*, *TGFB1*, *FN1*, and *ITGA5*) in clinically relevant subgroups. We found that mRNA expression of *NT5E*, *TGFB1*, *FN1*, and *ITGA5* is enriched in aggressive subtype of PDA (squamous/basal-like) (Figs 3e,f, S5a,c,e,g,i,j), and that PDA having high expression of those genes showed poor prognosis in the ICGC and/or in the TGCA cohorts (Figs 3g, S5b,d,f,h,k). On the other hand, mRNA expression of *C7* and *MUC1* was downregulated in squamous/basal-like tumours (Figs 3h–i, S6c,d). Moreover, high expression of *C7* and *MUC1* identifies PDA patients that tended to have better prognosis (Figs 3k, S6b,e). These results demonstrate that the ODIs can be used a biological resource for the identification of genes associated to the progression of PDA.

### M2 macrophages accumulate during progression of disease

In addition to the expression of genes known to be associated to T cell disfunction, we also used mRNA profiles from ODI to explore cell signatures associated to T cell exclusion. Reduced intratumoural T cell infiltration can be due to presence of immunosuppressive population or lack of T cell priming^25,26^.

**Figure 4:**
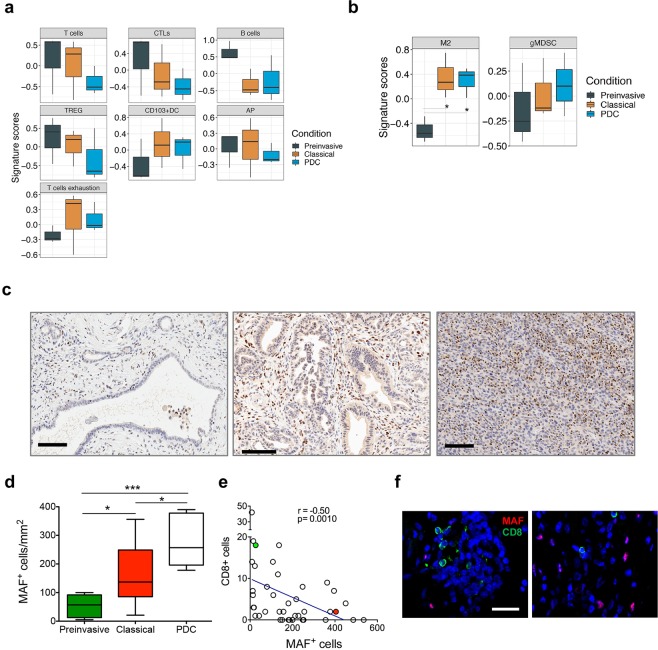
MAF positive macrophages accumulate during progression of ODI. (**a**,**b**) Box plots of signature scores in organoid derived isografts stratified according to the stage of disease. p values, Wilcoxon rank-sum test. Please refer to Supplementary Table 4 for details on genes used to define the specific gene signatures. **(c**) Immunohistochemical staining for Maf in tissues from mice bearing preinvasive lesions (left), classical tumours (middle), or poorly differentiated tumours (right). All tissues derive from B6-K3 organoid transplants. Scale bars, 100 µm. Quantification is provided in **(d)** as the average number of Maf positive nuclei per mm^2^ in preinvasive (n = 6), classical tumours (n = 10), and PDC (n = 7). From 3 to 5 individual areas per case were examined. Statistical associations were determined by Student’s t-test. *p < 0.05; ***p < 0.001. (**e**) Correlation between number of MAF positive and CD8 positive cells in mouse tumours considering all stages of disease (a total of 40 individual areas of 1 mm^2^ were considered) (Spearman r = −0.50, p = 0.010). Shown is the curve of linear fit correlation. **(f)** Immunofluorescence analysis for MAF (red) and CD8 (green), in mouse classical tumour tissues indicated as red or green circles in (**e**). Nuclei were counterstained with DAPI (blue). Scale bar, 20 µm. TREG, regulatory T cells; CTLs. Cytotoxic T lymphocytes; M2, anti-inflammatory macrophages; AP, antigen presentation; DC, dendritic cells; gMDSC, granulocytic-myeloid derived suppressor cells.

First, we explored gene signatures of antigen presentation and processing and found that only reduction of a B cells signature, in the comparison classical tumours vs preinvasive lesions, had a trend towards significance (p = 0.081) (Fig. 4a). This suggests that same mechanisms are likely operating very early in preinvasive lesions and persist throughout tumour development.

We then used expression profiles to examine two major populations known to reduce intratumoural T cell infiltration, namely granulocytic myeloid-derived suppressor cells (g-MDSC) and anti-inflammatory (M2) macrophages.

By flow-cytometric analysis we have already showed that granulocytes and macrophages were present as early as in PanIN-like lesions and persisted in established tumours without further expansion. Expression profiles and cell-type specific signatures (Supplementary Table 4) were used to evaluate whether signatures of immunosuppressive granulocytes and/or macrophages were enriched in advanced stages of ODI. We found a significant increase in signatures associated to immunosuppressive macrophages during progression from preinvasive lesions to invasive carcinomas (p = 0.01, Fig. 4b). To corroborate this finding, we orthogonally validated the increase in M2 during tumour progression in ODI by immunohistochemistry using a known marker of M2 macrophages, the transcription factor Maf (Fig. S7a)^32,38,39^. Immunohistochemical staining for Maf confirmed that M2 macrophages accumulated during progression in ODI (Fig. 4c,d) and inversely correlated to infiltration of CD8^+^ T cells (Fig. 4e,f).

### MAF expressing macrophages accumulates during progression of human PDA

**Figure 5:**
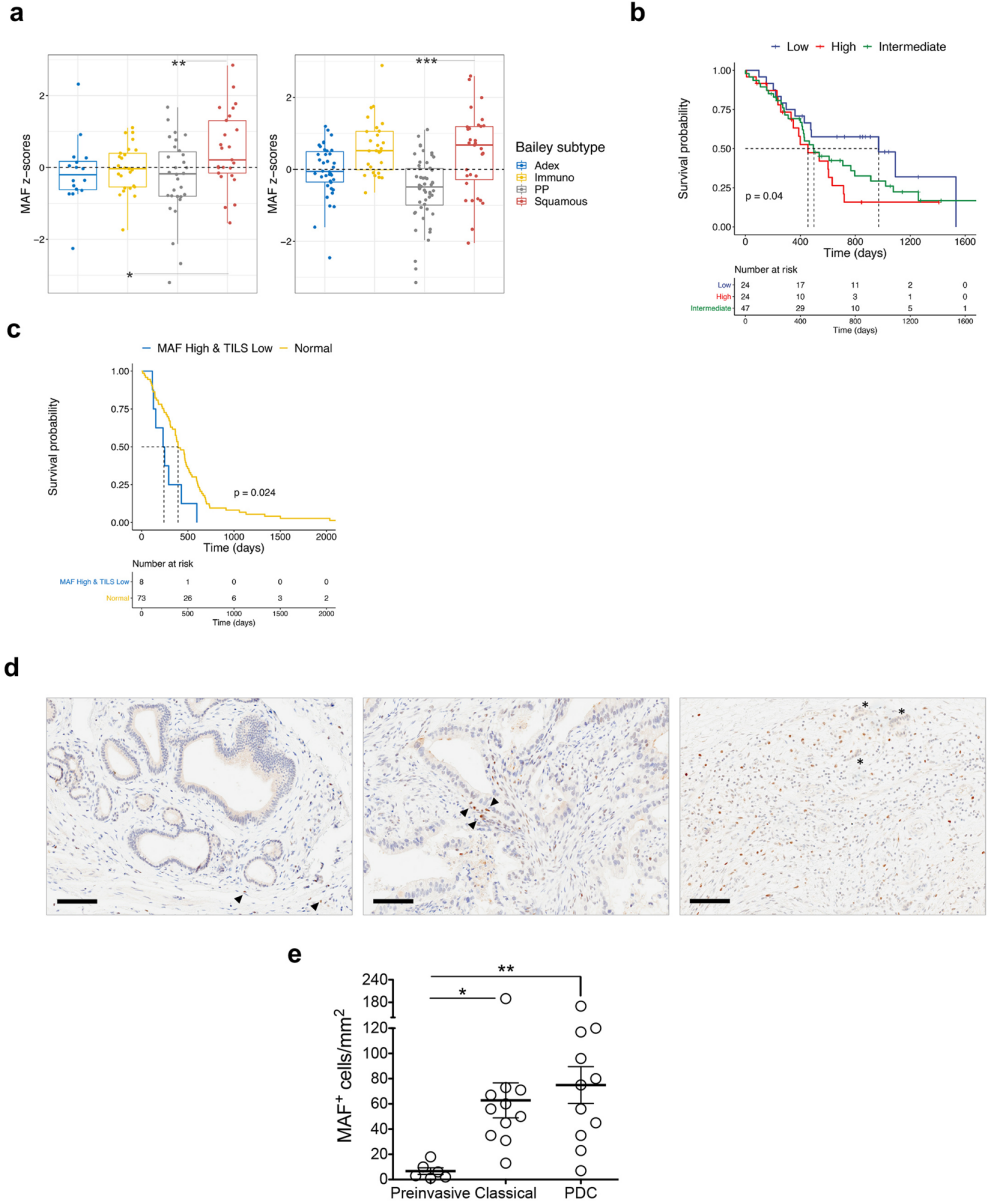
MAF is overexpressed in the aggressive subtype of PDA. (**a**) Box plot showing the MAF Z-score score stratified by Bailey subtypes in the ICGC-(left) and TCGA (right) cohorts. ***p < 0.001; **p < 0.01; *p < 0.05 as determined by Wilcoxon rank-sum test. **(b)** Kaplan–Meier analysis comparing survival of patients in the ICGC cohort having high, intermediate or low expression of *MAF*. p, Log-rank (Mantel-Cox) test. **(c)** The association between the CTL level and overall patient survival for PDA tumours with different MAF levels. For each tumour in the TCGA, the infiltration of cytotoxic T lymphocytes or Tumour-infiltrating lymphocytes (TILs) was estimated as the average expression level of GZMB, GZMA, PRF1, and CD8A. Kaplan–Meier analysis compares the survival of patients with low TILs and high expression. p, Log-rank (Mantel-Cox) test. (**d**) Immunohistochemical staining for MAF in human tissues distinguished in preinvasive lesions (left), classical tumours (middle), or poorly differentiated tumours (right). Scale bars, 100 µm. Quantification is provided in **(e)** as the average number of MAF positive nuclei per mm^2^ in preinvasive (n = 6), classical tumours (n = 11), and poorly differentiated carcinomas (PDC, n = 11). From 5 to 6 individual areas were examined per case. Statistical associations were determined by Student’s t-test. *p < 0.05; **p < 0.01.

To translate our results in the human disease, we first sought to assess the expression of *MAF* in relation to PDA subtypes and found that *MAF* is enriched in the subtypes dominated by macrophage signatures (Immunogenic and Squamous/basal-like) in the TCGA cohort and in the Squamous subtype in the ICGC cohort (Figs 5a and S7b)^6^. In the PDA cohort of the ICGC, patients with high expression of *MAF* has worst prognosis (Fig. 5b), and patients with high expression of *MAF* and low level of a signature of tumour-infiltrating lymphocytes had worst prognosis in the TCGA cohort (Fig. 5c). We then evaluated whether M2 macrophages were accumulating during progression in human tissues. Therefore, we selected 6 preinvasive lesions (2 PanINs and 4 IPMNs), 11 well-to-moderately differentiated PDA, and 11 tumours defined as squamous/Poorly differentiated based on the expression of known squamous cell markers, namely CK5 and p63^40^ (Fig. S7c). Consistently with observations in ODIs, macrophages expressing MAF were enriched in tumours compared to preinvasive lesions (Fig. 5d,e), although no statistically significant difference was observed between classical tumours and poorly differentiated tumours.

## Discussion

Increasing evidence suggests that cancer cell programs driven by specific genetic events dictate the immune contexture of PDA^6,14^. However, recent studies conducted in GEMM have demonstrated that the selective targeting of myeloid cell populations influence gene expression programs of cancer cells^11,12^. It is becoming clear that the ability of conducting integrative and dynamic genotypic-immunophenotypic analyses is a fundamental requirement for the identification of undisclosed therapeutic strategies that effectively elicit anti-tumour immunity. Here, we tracked the immunoevolution of PDA in a genetically-defined and transplantable model of mouse pancreatic tumour organoids that recapitulates the progression of the disease from early preinvasive lesions to metastatic carcinomas. We demonstrated that leukocytes heavily infiltrated the pancreas as early as in preinvasive lesion, which is in line with previous observation in autochthonous model of the disease^15^. Myeloid cells, and in particular granulocytes, were the most represented immune subpopulation in preinvasive lesions, which outnumbered lymphocytes during progression. We showed that the expansion of granulocytes within the tumour-infiltrating leukocytes compartment was accompanied by increased level of serum G-CSF and dramatic decrease of T cell infiltration, which suggest mechanisms of T cell exclusion. However, it should be noted that the absolute number of infiltrating CD8^+^ T cells was scarce even in preinvasive lesions. Progression from preinvasive lesions to overt cancers was also associated to increased expression of potent immunosuppressive genes, among which *Nt5e* and *Tgfβ1* have been previously shown to induce T cell dysfunction^29–31,34^. Notably, we found that the expression of the two genes was enriched in aggressive subtype of PDA and negatively correlated with patients’ survival. Along with the identification of *C7* and *MUC1*, whose high expression indicated better survival, our data demonstrates that the ODI can be used a biological resource for the identification of clinically-relevant biomarkers in PDA. In line with previous observation^15^, we also found that macrophages were present at preinvasive stage and persisted throughout PDA. However, we found a significant expansion of M2 macrophages during progression of the disease. Polarization of macrophages towards M2 phenotype is supported by an immunosuppressive cytokine milieu composed of tumour- and stromal-derived factors that include TGF*β*, IL-6 and IL-10^32,41–43^. Consistently, we found elevated serum level of IL-6 and IL-10 as lesions progressed over time. MAF expressing M2 macrophages accumulated during progression and inversely correlated with infiltration of CD8^+^ T cells in mouse tumours, suggesting that they might be the prominent immune cell type to mediate T cell exclusion. We confirmed that expression of *MAF* is higher in PDA subtypes dominated by macrophage-related signatures, in particular in the squamous subtype, and negatively correlated with patients’ survival in the ICGC cohort. Among PDA tumours of the TCGA cohort, low TILs levels indicated a worse patients’ survival but only when *MAF* had lower expression, which is in line with the antagonist interaction between Maf^+^ macrophages and CD8^+^ T cells that we found in ODI. Although we only looked at few cases, density of MAF expressing macrophages was significantly higher in human PDA tissues compared to preinvasive lesions, which included PanINs and IPMNs. Of the common PDA drivers, mutations *Kras* and *Trp53* were the only detectable oncogenic events across lesions at different stages of the disease with the exception of the LOH affecting *Trp53* in poorly-differentiated and metastatic tumours. However, considering that we only looked at a limited genomic space (19 commonly mutated genes in PDA and peripancreatic tumours), we cannot rule out the possibility that progression of this model is associated with accumulation of other genomic events. Mouse organoids can be genetically manipulated *ex vivo*, which provides the potential of creating models for interrogating effects of specific genotypes on immune contexture of PDA. Overall, our results show that, in ODI, progression of disease is associated to an increased immunosuppressive microenvironment that outweighs antitumour cellular immunity, thereby likely contributing to disease progression. We also showed that MAF expressing macrophages are prominent myeloid cells population in both mouse and human PDA, which warrants future investigation for potential therapeutic intervention.

## Materials and Methods

### Patients’ samples

Pancreatic cancer tissues were obtained from patients undergoing surgical resection at the University Hospital Trust of Verona. Ethics committee approval was obtained at *University of Verona*, *Italy:* approval number 1885 from the Integrated University Hospital Trust (AOUI) Ethics Committee (Comitato Etico Azienda Ospedaliera Universitaria Integrata). Written informed consent from the donors for research use of tissue in this study was obtained prior to acquisition of the specimen. Samples were confirmed to be tumour or normal based on pathological assessment. All experiments were conducted in accordance with relevant guidelines and regulations. Human formalin-fixed paraffin-embedded (FFPE) tissues were used for immunohistochemical staining. A total of 28 tissue specimens were used, including: 6 preinvasive lesions (2 PanINs and 4 IPMNs), 11 well differentiated PDA (defined as “classical”), 11 squamous/poorly differentiated tumours.

### Mice

*Trp53*^*+/LSL-R172H*^, *Kras*^*+/LSL-G12D*^ and *Pdx1-Cre* strains on a C57Bl/6 background were interbred to obtain *Pdx1-Cre; Kras*^*+/LSL-G12D;*^ *Trp53*^*+/LSL-R172H*^ (KPC) mice^16^. Six- to 8-weeks old C57Bl/6 J (B6J) mice were purchased from Charles River Laboratory. All animal experiments were conducted in accordance with procedures approved by CIRSAL at University of Verona (approved project 655/2017-PR).

### Mouse pancreatic ductal organoid culture

Mouse pancreatic tumour cells were isolated from the tumour bulk of mice older than 8 weeks as previous described^17,19^. Briefly, mouse pancreatic tumours were minced and digested by enzymatic dissociation with 5 mg/ml Collagenase type XI (Gibco), 1 mg/ml Dispase (Gibco), 1% FBS (Gibco) in DMEM medium (Gibco) at 37 °C for a maximum of 16 hrs. Isolated material was incubated with TrypLE (Gibco) at 37 °C for 10 min, embedded into growth factor-reduced Matrigel (Corning), and cultured in mouse complete medium (AdDMEM/F12 (Gibco) supplemented with 1% penicillin/streptomycin (Gibco), 1% GlutaMAX (Gibco), 10 mM HEPES (Gibco), 1:50 B27 supplement (Gibco), 1.25 mM N-Acetylcysteine (Sigma), 10% (vol/vol) Rspo1-conditioned media, 10 mM Nicotinamide (Sigma), 10 nM recombinant human-gastrin I (Tocris), 50 ng/ml recombinant mouse EGF (Gibco), 100 ng/ml recombinant human FGF10 (Peprotech), 0.5 µM A83-01 (Tocris), and 100 ng/ml recombinant human Noggin (Peprotech)). Mouse complete medium was changed twice a week, and cultures were split upon the attainment of dense culture. Passage was performed in a 1:4–1:8 split ratio.

### Targeting sequencing

High-coverage sequencing of organoid cultures and tissues from organoid transplants was performed using an AmpliSeq custom panel (ThermoFisher) targeting all exons of 19 genes frequently mutated in pancreatic and peripancreatic tumours (detailed list of genes in Supplementary Table 1). For each reaction, 20 ng of DNA were used, and the quality of the resulting libraries evaluated by the Agilent 2100 Bioanalyzer on-chip electrophoresis (Agilent Technologies). Sequencing runs were performed on the Ion Proton (PI, ThermoFisher) loaded with Ion PI Chip v2. Base calling, alignment to the mm10 mouse reference genome, and variant calling were done using the Torrent Suite Software v.5.0 (ThermoFisher). Alignments were visually verified with the Integrative Genomics Viewer (IGV)^44^. Targeted sequencing data were also used to estimate loss of heterozygosity (LOH).

### Pancreatic organoid transplantation

For the orthotopic transplantation of mouse organoids, recipient mice were anesthetized with isoflurane. Organoids (1 × 10^6^ cells/mouse) were recovered from Matrigel using ice-cold Cell Recovery Solution (Corning) for 30 min, and then mechanically dissociated into small fragments through fire-polished glass pasteur pipettes. Before transplantation, organoids were resuspended in 50 µl of a 2:3 dilution of Matrigel and cold PBS. An incision was made in the left abdominal side at the level of the spleen. Organoids were injected into the tail region of the pancreas using insulin syringes (BD micro-fine 30 G). The injection was considered successful by the development of bubble without signs of leakage. The peritoneum was sutured with short-term absorbable suture (Vetsuture), and the skin was closed with wound clips (CellPoint Scientific Inc.). Mice were sacrificed at the indicated time points. Monitoring of tumour growth was performed as previously described^18^. Briefly, following weekly manual palpation starting 10 days following transplantation, tumour-bearing mice were subjected to high-contrast ultrasound screening using the Vevo 2100 System with a MS250, 13–24 MHz scanhead (Visual Sonics, Inc, Amsterdam, NL).

### Flow cytometry

The tumour flow cytometry-based immuno phenotype was performed according to already published protocols^45^. Briefly, tumours were minced and digested for about 1 hr at 37 °C under continuous mixing with a digestive mix containing 1 mg/mL collagenase IV, 0.1 mg/mL hyaluronidase, and 30 U/mL DNAse in RPMI 1640, all purchased from Sigma-Aldrich. The cell suspension was separated from the undigested material using a 70-μm cell strainer (Corning). On the contrary, the analysis of circulating leukocytes was performed using splenocytes collected by mechanical disruption of the tissue followed by red blood cell lysis with the ACK buffer (Lonza). One million of cells were incubated with anti-mouse CD16/32 (Biolegend) and subsequently stained with the following antibodies according to the vendor’s instructions: CD3 (17A2, Thermo Fisher Scientific), CD4 (GK1.5, Thermo Fisher Scientific), CD8a (53.6.7, Thermo Fisher Scientific), F4/80 (A3-1, Bio-Rad), MHC2 (M5/114.15.2, Thermo Fisher Scientific), CD11b (M1/70, Thermo Fisher Scientific), B220 (RA3-6B2, Thermo Fisher Scientific) CD11c (N418, Thermo Fisher Scientific), LY6C (HK1.4, Biolegend), LY6G (1A8, Biolegend), CD45 (30F11, Biolegend), Samples were acquired on a FACS Canto II (BD Biosciences) and analyzed with FlowJo software (FlowJo LLC).

### Histology and immunohistochemistry

Tissues were fixed in 10% neutral buffered formalin and embedded in paraffin. Sections were subjected to Hematoxylin and Eosin, and Masson’s Trichrome staining as well as immunohistochemical staining. The following primary antibodies were used for immunohistochemical staining and established procedures^46^: F4/80 (ab6640, Abcam), 1:100; MAF (sc-7866, clone M-153, Santa Cruz) 1:200; CD8 (14-0808-82, Thermo Scientific) 1:2000; CD206 (ab64693, Abcam) 1: 200; CK5 (XM26, Novocastra) 1:100; p63 (DAK p63, DAKO) 1:50. Staining quantification was performed using ImageJ. For immunofluorescence staining, paraffin slides were deparaffinized and subjected to antigen retrieval using citric acid buffer (pH 6). Primary antibodies (1:200 MAF; 1:750 CD8) were then applied overnight at 4 °C, and then revealed by incubation with secondary antibodies conjugated to fluorophores. To reduce background nonspecific staining, slides were immersed in 0.1% Sudan Black B (Carlo Erba) in 70% ethanol for 20 min, and then washed with PBS. Nuclei were stained with DAPI (Sigma). Images were captured on a Zeiss AxioObserver Z1 inverted fluorescence microscope (Zeiss).

For multiplex immunofluorescence staining, we used the Opal Multiplex IHC Kit (Akoya) and the following antibodies: CD8α (98941, Cell Signaling) 1:100; FoxP3 (12653, Cell Signaling) 1:100. Briefly, FFPE sections were deparaffinized and then subjected to several sequential of microwave treatment and staining. Each includes antigen retrieval by heat-induced epitope retrieval using citrate buffer (pH6), a protein blocking followed by primary antibody, introduction of Secondary-HRP, and incubation with Opal Fluorophore for 10 min at room temperature. After all sequential staining reactions, sections were counterstained with DAPI (Vector lab). Slides were scanned by Leica TCS SP5 laser scanning confocal (Leica) with 80 × objective magnification and digitalized by the Leica Application Suite X (LAS X) software. Immunofluorescence images were quantified using five fields per tumour.

### Multiplex cytokines profiling

Mouse plasma specimens were analyzed using a mouse multiplex ELISA kit according to the manufacturer instructions (Bio-Rad Laboratories, Hercules, California, U.S.A.). All samples were tested for the expression of circulating interleukin (IL)1α, IL-1β, IL-2, IL-3, IL-4, IL-5, IL-9, IL-12p40, IL-12p70, IL-13, CCL11 (eotaxin), granulocyte-macrophage colony-stimulating factor (GM-CSF), interferon (IFN)γ, monocyte chemoattractant protein (MCP-1; CCL2), macrophage inflammatory protein 1α (MIP-1α; CCL3), macrophage inflammatory protein 1*β* (MIP-1*β*; CCL4), CCL5 (RANTES), CXCL1 (KC), tumour necrosis factor (TNF)α. Median fluorescence intensities were collected on a Bio-Plex 200 instrument, using Bio-Plex Manager software version 6.2. Standard curves for each cytokine were generated using the premixed lyophilized standards provided in the kits. Median fluorescent intensities were transformed into cytokines concentrations by 5-point-regression.

### mRNA profiling and qRT-PCR

Total RNA was extracted from formalin fixed paraffin embedded (FFPE) tissues using the RecoverAll™ Total Nucleic Acid Isolation Kit for FFPE (ThermoFisher). A concentration of at least 10 ng/*μ*l was achieved for each sample. Purity of RNA was suitable when A260/A280 ratio was over 1.7 and A260/A230 ratio was over 1.8. Expression analysis was performed using Nanostring Mouse PanCancer Immune Profiling Kit (Diatech XT-CSO-MIP1-12). For FFPE tissues 150 ng of RNA was used according to the manufacturer’s protocol. The matrix of genes counts was then used for differential expression analysis with *DESeq2* Bioconductor package^47^. *DESeq2* was used in combination with *RUVSeq*^48^ in order to control housekeeping genes expression and for normalization purposes. A batch factor of variation was calculated from the expression of the housekeeping genes, and such factor was then added to the *DESeq2* design formula. Gene set variant analysis was performed with the *GSVA* Bioconductor package with the following parameters: *method* = *‘gsva’*, *mx*.*diff* = *TRUE*, *kcdf* = *‘Gaussian’*^49^. The list of gene sets used, and associated references is available in the Supplementary Table 4.

For qPCR analyses, 500 ng of total RNA was reverse transcribed using SuperScript^®^ VILO^TM^ cDNA Synthesis Kit (Life Technologies) in a volume of 20 µl according to the manufactures’ instructions. Samples were diluted to a final concentration of 10 ng/µl. TaqMan was performed in triplicate using 20 ng of cDNA and the following TaqMan^®^ probe (TaqMan^®^ Gene Expression Assay): *Nt5e* (Mm00501910_m1); *Arg1* (Mm00475988_m1); *Tgfb1* (Mm01178820_m1); *C7* (Mm01297045_m1); and *Lcn2* (Mm01324470_m1). *Hprt1* was used as reference gene. Relative gene expression quantification was performed using the ΔΔCt method with the Sequence Detection Systems Software, Version 1.9.1 (Applied Biosystems).

### Data mining

For data mining and pancreatic cancer subtypes stratifications we used two different datasets. The first dataset represents the PACA-AU cohort of the ICGC consortium, downloaded from the supplemental material of the corresponding publication^6^. This dataset contains normalized expression values (TMM normalized using *edgeR* Bioconductor package, converted to CPM and log2 transformed) of 96 pancreatic cancer patients; for subtypes stratification, z-scores were calculated for each gene. Associated clinical data were downloaded from https://dcc.icgc.org/releases/current/Projects/PACA-AU. The second dataset represents the TCGA-PAAD cohort, downloaded from http://firebrowse.org/?cohort = PAAD, which consists of the RNA-Seq gene expression profile of 178 pancreatic cancer patients. According to other publications that disputed the purity of some samples, we restricted the number to 148 assured samples. The grouping of the samples in Bailey’s and Moffitt’s subtypes^7^ was performed with the *GSVA* Bioconductor package with the same options as above. The gene sets used for the stratification were retrieved from the original publications.

### Statistical analysis

Differences between experimental conditions were tested using Student’s t-test or Wilcoxon rank-sum test, according to the normality of samples’ distribution verified with a Shapiro-Wilk test. A p-value < 0.05 was considered statistically significant. Spearman’s rho correlation coefficient method was used to assess correlation between two variables. Survival analyses were performed using the computing environment *R*^50^ and the packages *survival*^51^, for fitting the model, and *survminer*^52^ for plotting. Overall survival data were obtained from the same link reported above. Median survival was estimated with the Kaplan-Meier method and the difference was tested using the log-rank (Mantel-Cox) test. For stratifying survival, gene expression data were divided into quartiles.

## Supplementary information

Supplementary Figures

Supplementary Tables

## Data Availability

Supporting data and protocols are made available without restrictions.
