# Supplementary material for: Immunoevolution of mouse pancreatic organoid isografts from preinvasive to metastatic disease
Source: Sci Rep. 2019 Aug 22;9:12286. doi: 10.1038/s41598-019-48663-7 (PMC6706454; doi:10.1038/s41598-019-48663-7)

**Immunoevolution of mouse pancreatic organoid isografts from preinvasive to metastatic disease**

Dea Filippini^1^*, Sabrina D’Agosto^2^*, Pietro Delfino^1^, Michele Simbolo^1^, Geny Piro^3¶^, Borislav Rusev^2^, Lisa Veghini^1^, Cinzia Cantù^2^, Francesca Lupo^1^, Stefano Ugel^4^, Francesco De Sanctis^4^, Vincenzo Bronte^4^, Michele Milella^3^, Giampaolo Tortora^3¶^, Aldo Scarpa^1,2^, Carmine Carbone^3¶§^ & Vincenzo Corbo^1,2§^^

^1^Department of Diagnostic and Public Health, University of Verona

^2^ARC-Net Research Centre, University of Verona

^3^Department of Medicine, Section of Medical Oncology, University of Verona

^4^Department of Medicine, Section of Immunology, University of Verona

^¶^ Present address: Fondazione Policlinico Universitario A. Gemelli IRCCS, Rome, Italy; Università Cattolica Del Sacro Cuore, Rome, Italy

*share first authorship

^§^senior authors

^correspondence to Vincenzo Corbo, Department of Diagnostic and Public Health, ARC-Net Research Centre, University of Verona, Piazzale L.A. Scuro, 10, 37134, Verona, Italy. Email: [vincenzo.corbo@univr.it](mailto:vincenzo.corbo@univr.it).

**SUPPLEMENTARY INFORMATION**

**Supplementary Figures File**

- Figure S1. Establishment and characterization of organoid cultures from mouse pancreatic tissues
- Figure S2. Analysis of major immune populations infiltrating tumours from organoid isografts
  Figure S3. Serum levels of cytokines during progression of the organoid-derived isografts
- Figure S4. Changes in the expression of immune-related genes during progression of organoid-derived isografts
- Figure S5. Expression and prognostic value in human PDA of immune-related genes upregulated during progression of ODIs
- Figure S6. Expression and prognostic value in human PDA of immune-related genes downregulated during progression of ODIs.
- Figure S7. Expression of MAF in human PDA

**Supplementary Tables**

- Table S1. Genes included in the targeted sequencing panel
- Table S2. List of differentially expressed genes
- Table S3. List of genes included in the Venn diagram of Figure S4
- Table S4. Gene signatures used for the analysis in Figure 4

**SUPPLEMENTARY FIGURE LEGENDS**

**Supplementary Figure 1. Establishment and characterization of organoid cultures from mouse pancreatic tissues. (a)** Representative Hematoxylin & Eosin staining of the mouse pancreatic ductal adenocarcinomas (from left to right, B6-K1, -K2, -K3) used to generate organoid cultures. **(b)** Representative brightfield images of mouse organoid cultures established from normal tissue (left) and primary tumours (right). Scale bars in (a), 200 µm; scale bars in (b), 100 µm. **(c)** Targeted (n = 19) sequencing analysis of tumour (B6-K1/3) and normal (B6-N1/3) organoids. The status of the 4 more commonly mutated PDA genes is shown. Color key indicates type of mutation. **(d)** Workflow of the transplantation experiment with 3 different mouse tumour organoid cultures. 1x10^6^ cells were orthotopically transplanted into individual syngeneic mice (n = 30) and growth initially monitored by weekly palpation. Upon mass detection, growth was monitored weekly by high-contrast ultrasonography. As indicated, mice were euthanized at different times from transplantation (m, months). **(e)** Hematoxylin & Eosin (H&E) and Masson’s trichrome (MT) staining of lesions from early orthotopic transplants of mouse organoid cultures (Preinvasive, 1 months from transplantation of B6-K2 organoids). Dashed lines indicate the areas magnified in insets. **(f)** Hematoxylin & Eosin (top panel) and Masson’s trichrome (bottom panel) staining of classical tumours (3 months from transplantation of B6-K3 organoids). Scale bars, 200 µm unless otherwise indicated. Magnification of selected areas (dashed lines) is provided in the insets.

**Supplementary Figure 2. Analysis of major immune populations infiltrating tumours from organoid isografts. (a)** Flow cytometry analysis of CD45^+^ cells in the pancreas of preinvasive (n = 5), classical tumours (n = 5), or PDC (n = 4). Data are displayed as scatter dot plot with SD (standard deviation) **(b)** Flow cytometry analysis of CD45^+^ cells in the spleen of tumour-free mice (naïve, n = 3) and mice bearing lesions (black = PDC, green = preinvasive, red = classical). **(c)** Flow cytometry analysis of myeloid cells (CD45^+^CD11b^+^) cells in the pancreas of preinvasive, classical tumours, or PDC. **(d)** Flow cytometry analysis of myeloid cells (CD11b^+^) as percentage of CD45^+^ cells in the pancreas of preinvasive, classical tumours, or PDC. **(e)** Flow cytometry analysis of B cells (CD45^+^B220^+^) cells in the pancreas of preinvasive, classical tumours, or PDC. **(f)** Flow cytometry analysis of B cells (B220^+^) as percentage of CD45^+^ cells in the pancreas of preinvasive, classical tumours, or PDC. ODIs from B6-K1, B6-K2, and B6-K3 organoids are identified by circles, triangles, and squares, respectively**. (g)** Dual immunofluorescence for Foxp3 (red) and CD8 (green) in tissues from mice bearing preinvasive, classical tumour, and PDC. Scale bar, 20 µm. Magnification is provided in insets. Nuclei were counterstained with DAPI (blue). All tissues were from B6-K1 transplants. Quantification is provided in (**h**) as the average number of Foxp3^+^ cells per field of visualization (FOV, 80X magnification) in preinvasive (n = 5), classical tumours (n = 5), and PDC (n = 5). A minimum of 3 areas per case were examined. (**i**) Ratio of CD8^+^ to Foxp3^+^ T cells from (h).

Statistical associations were determined by Student’s t-test. *, p < 0.05; **, p < 0.01.

**Supplementary Figure 3. Serum levels of cytokines during progression of the organoid-derived isografts.** Multiplex bead-based human cytokine assay for serum detection of circulating factors in mice bearing preinvasive lesions (n = 5), classical tumours (n = 6) and poorly differentiated tumours (PDC, n = 5). Mean and SEM in pg/mL are shown.

**Supplementary Figure 4. Changes in the expression of immune-related genes during progression of organoid-derived isografts.** Heatmaps showing changes in the expression pattern of the 30 most differentially expressed immune-related genes in the comparison between: classical and preinvasive lesions (**a**); PDC and preinvasive lesions (**b**); PDC and classical tumours (**c**); tumours (classical and poorly differentiated) and preinvasive lesions (**d**). **(e)** Venn diagram illustrates shared and unique genes upregulated and downregulated in the comparison of expression profiles of classical tumours (yellow) and PDC (blue) compared to preinvasive lesions; please refer to Supplementary Table 3 for details. **(f)** qRT-PCR for indicated genes in preinvasive lesions (n = 3), classical tumours (n = 3), and PDC (n = 3).

**Supplementary Figure 5 Expression and prognostic value in human PDA of immune-related genes upregulated during progression of ODIs. (a)** Box plot showing the *NT5E* Z-score score stratified by Bailey (left) or Moffitt subtypes (right) in the TCGA-PDA cohort. ****, p << 0.001 as determined by Wilcoxon rank-sum test. **(b)** Kaplan–Meier analysis comparing survival of patients in the TCGA-PDA cohort having either high or low expression of *NT5E*. p, Log-rank (Mantel-Cox) test. **(c)** Box plot showing the *TGFB1* Z-score score stratified by Bailey (left) or Moffitt subtypes (right) in the ICGC-PDA cohort. ****, p << 0.001 as determined by Wilcoxon rank-sum test. **(d)** Kaplan–Meier analysis comparing survival of patients in the ICGC cohort having either high or low expression of *TGFB1*. p, Log-rank (Mantel-Cox) test. **(e)** Box plot showing the *TGFB1* Z-score score stratified by Bailey (left) or Moffitt subtypes (right) in the TCGA-PDA cohort. ***, p < 0.001 as determined by Wilcoxon rank-sum test. **(f)** Kaplan–Meier analysis comparing survival of patients in the TCGA cohort having either high or low expression of *TGFB1*. p, Log-rank (Mantel-Cox) test. **(g)** Box plot showing the *FN1* Z-score score stratified by Bailey (left) or Moffitt subtypes (right) in the ICGC-PDA cohort. ****, p << 0.001 as determined by Wilcoxon rank-sum test. **(h)** Kaplan–Meier analysis comparing survival of patients in the ICGC cohort having either high or low expression of *FN1*. p, Log-rank (Mantel-Cox) test. **(i)** Box plot showing the *FN1* Z-score score stratified by Bailey (left) or Moffitt subtypes (right) in the TCGA-PDA cohort. ****, p << 0.001 as determined by Wilcoxon rank-sum test. **(j)** Box plot showing the *ITGA5* Z-score score stratified by Bailey (left) in the ICGC-PDA cohort or Moffitt subtypes (right) in the TCGA-PDA cohort. ****, p << 0.001 as determined by Wilcoxon rank-sum test. (**k**) Kaplan–Meier analysis comparing survival of patients in the ICGC cohort having either high or low expression of *ITGA5*. p, Log-rank (Mantel-Cox) test.

**Supplementary Figure 6**. **Expression and prognostic value in human PDA of immune-related genes downregulated during progression of ODIs. (a)** Box plot showing the *C7* Z-score score stratified by Bailey (left) or Moffitt subtypes (right) in the TCGA-PDA cohort. **(b)** Kaplan–Meier analysis comparing survival of patients in the TCGA-PDA cohort having either high or low expression of *C7*. p, Log-rank (Mantel-Cox) test. **(c)** Box plot showing the *MUC1* Z-score score stratified by Bailey (left) or Moffitt subtypes (right) in the ICGC-PDA cohort. ****, p << 0.001 as determined by Wilcoxon rank-sum test. **(d)** Box plot showing the *MUC1* Z-score score stratified by Bailey (left) or Moffitt subtypes (right) in the TCGA-PDA cohort. ****, p << 0.001 as determined by Wilcoxon rank-sum test. **(e)** Kaplan–Meier analysis comparing survival of patients in the ICGC cohort having either high or low expression of *MUC1*. p, Log-rank (Mantel-Cox) test.

**Supplementary Figure 7. Expression of MAF in human PDA. (a)** Immunohistochemical staining for the macrophage marker F4/80, the M2 marker MAF and CD206 in serial sections of a PDA tissues showing co-localization of nuclear MAF and membrane CD206 staining. Scale bars, 50µm. **(b)** Box plot showing the *MAF* Z-score score stratified by Moffitt subtypes in the ICGC- (left) and TCGA-PDA (right) cohorts. **, p < 0.01 as determined by Wilcoxon rank-sum test. **(c)** Representative immunohistochemical staining of human PDA tumours for the epithelial marker PDX1 and the squamous-cell markers CK5 and p63.


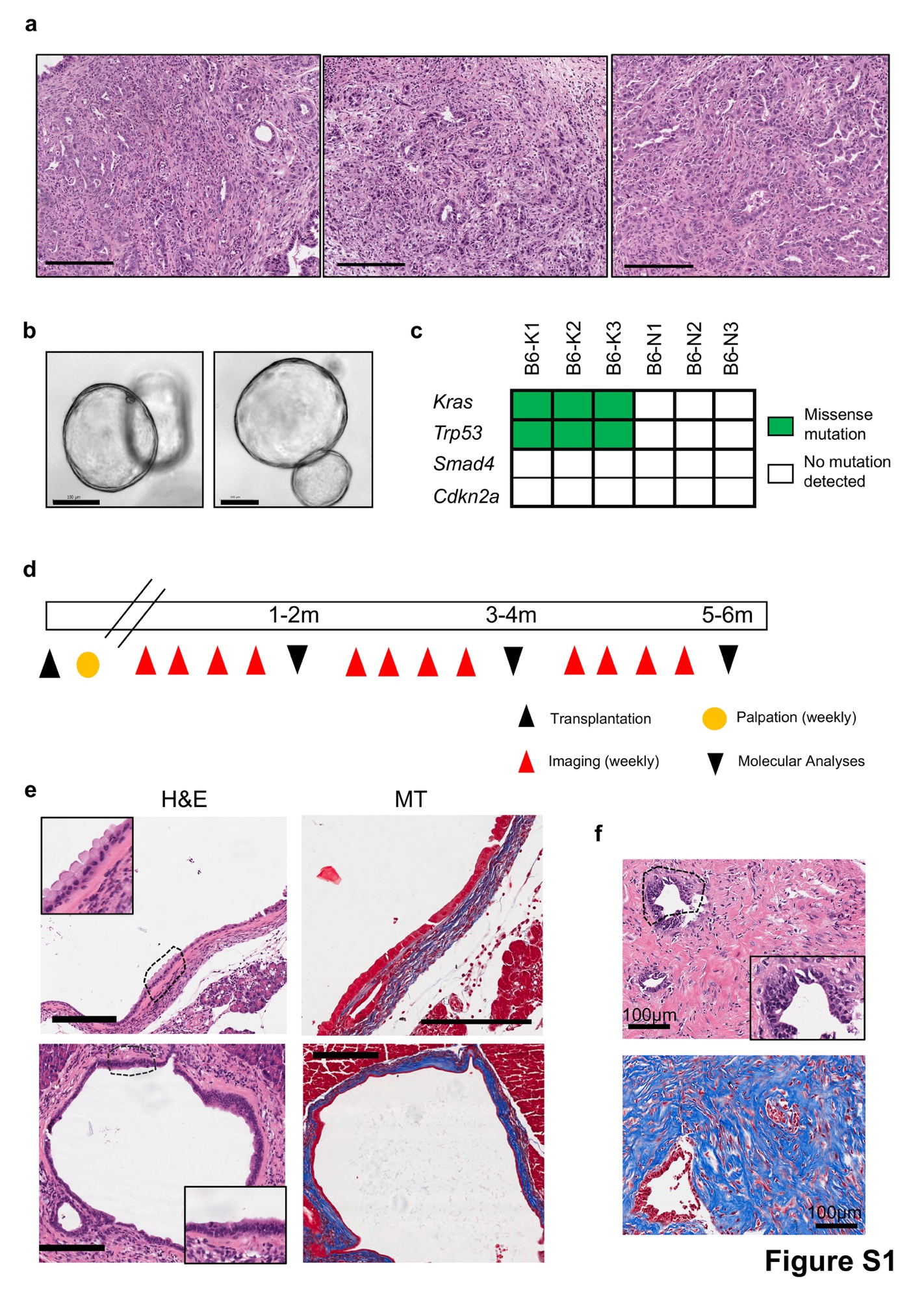


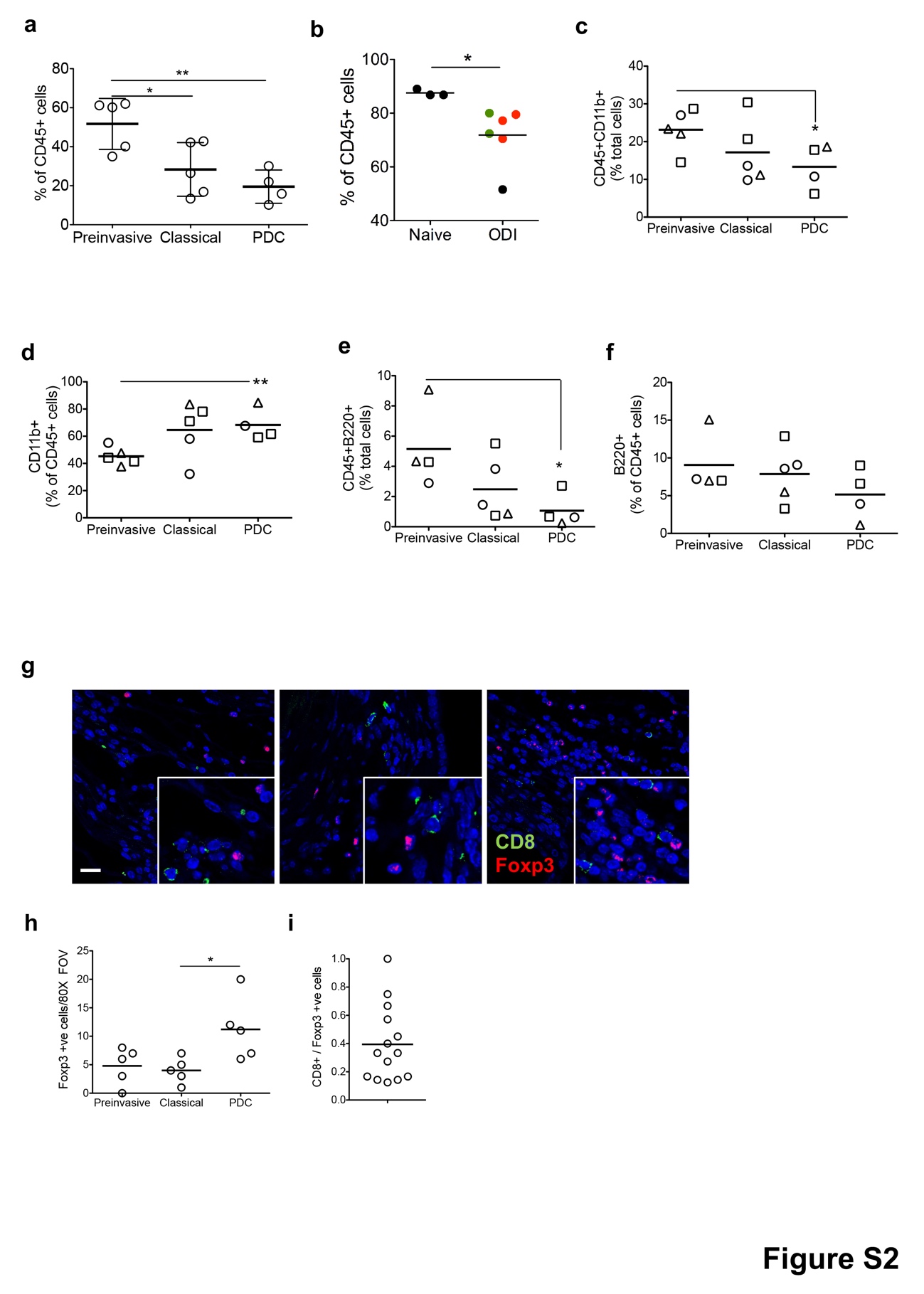


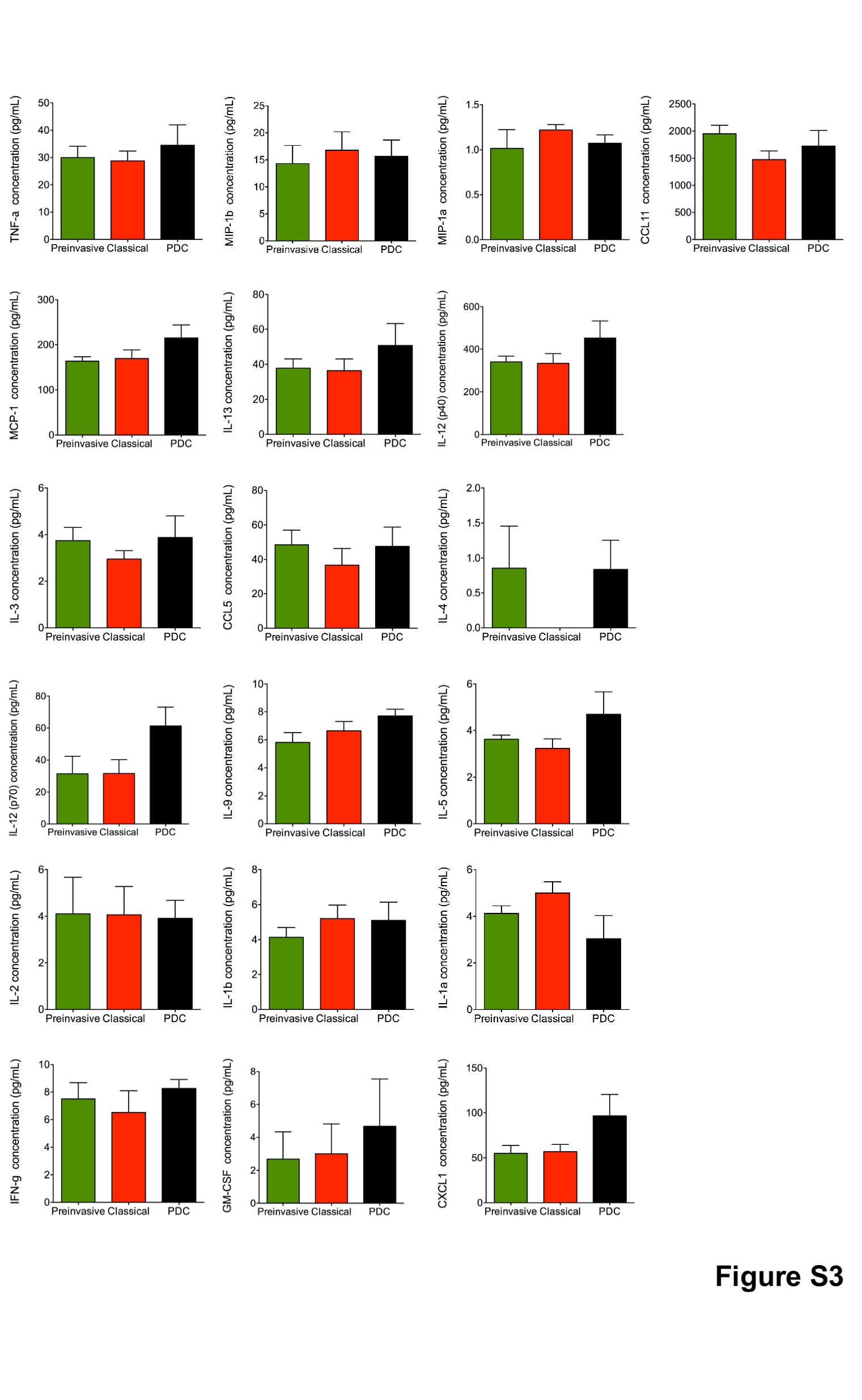


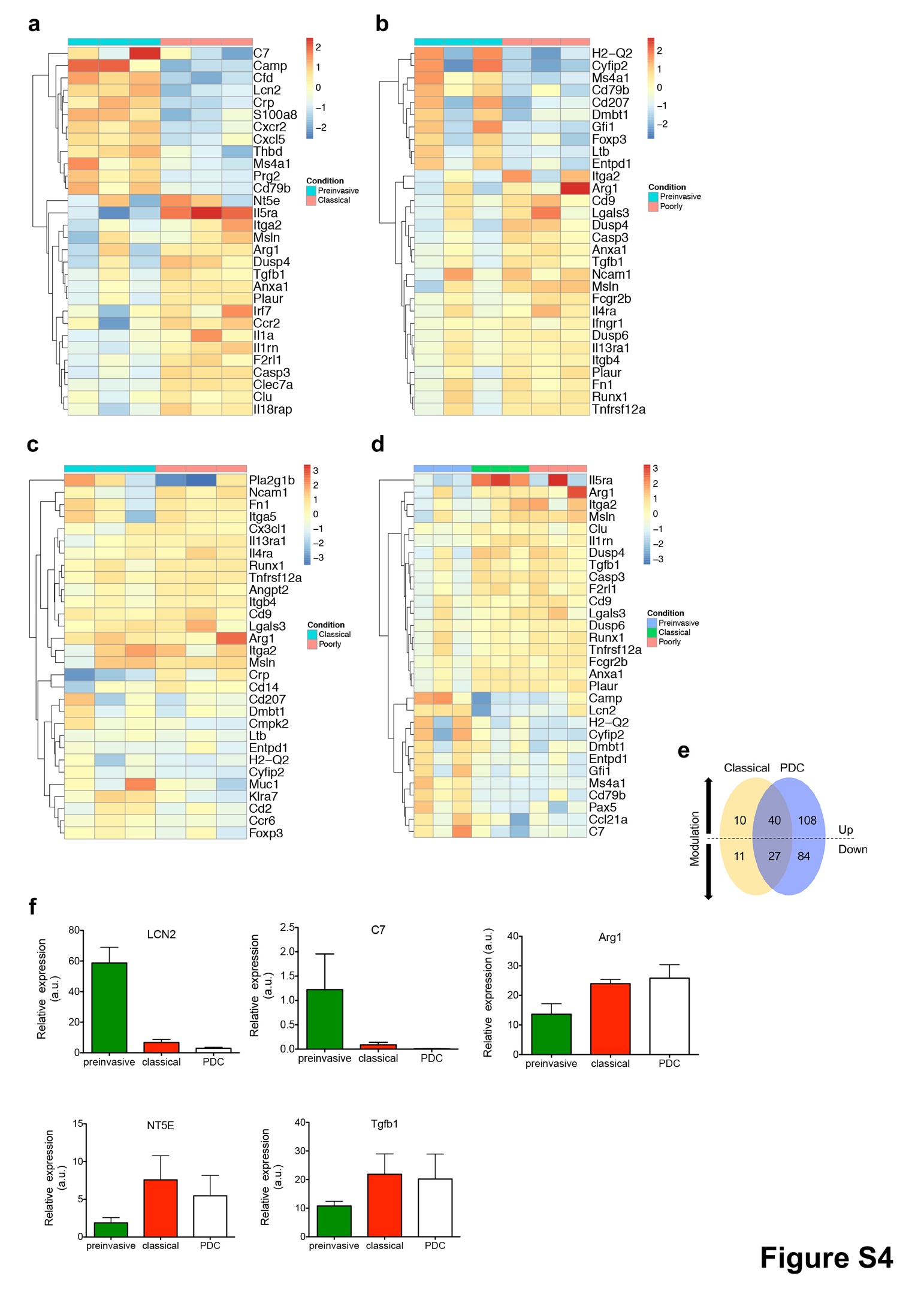


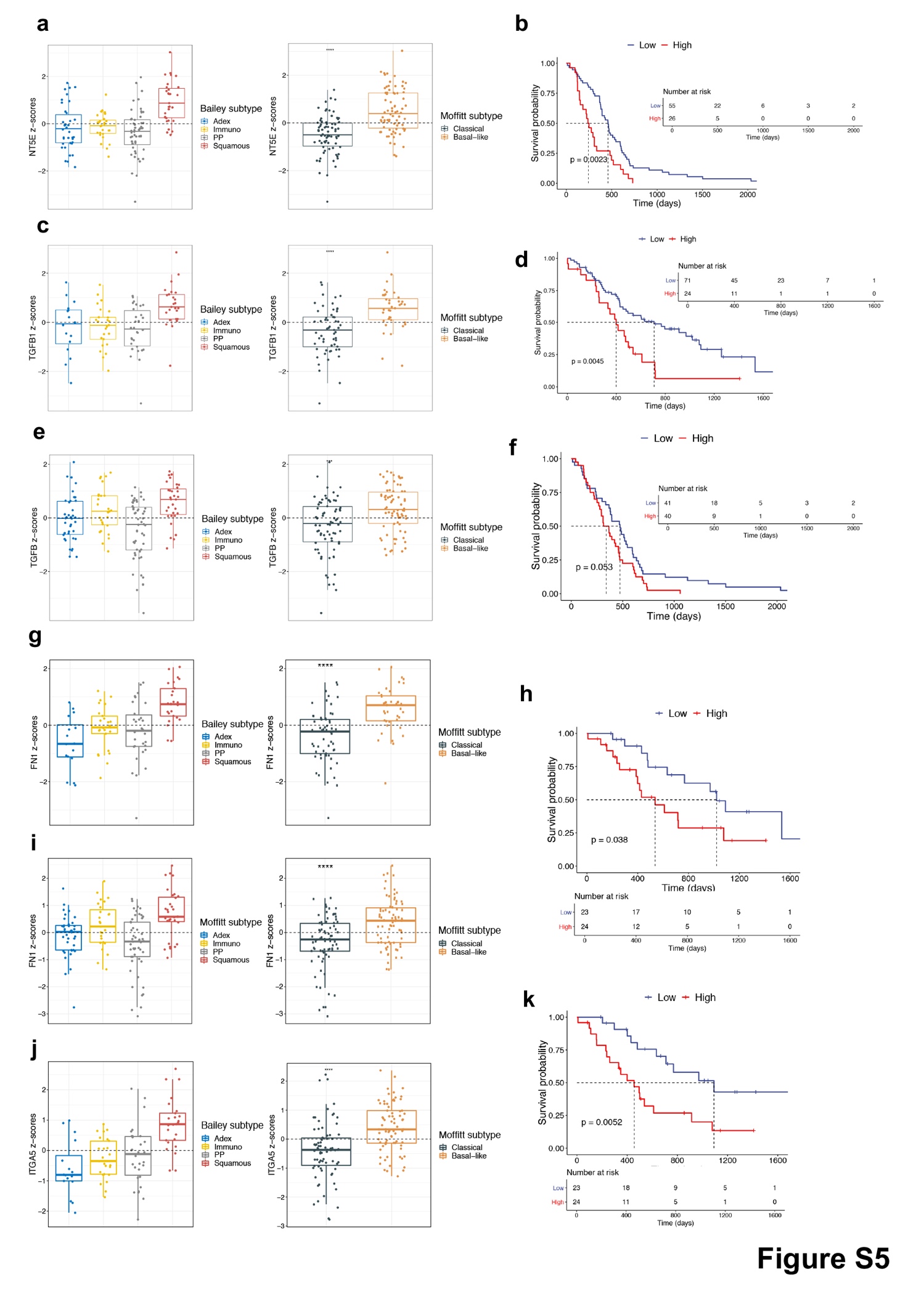


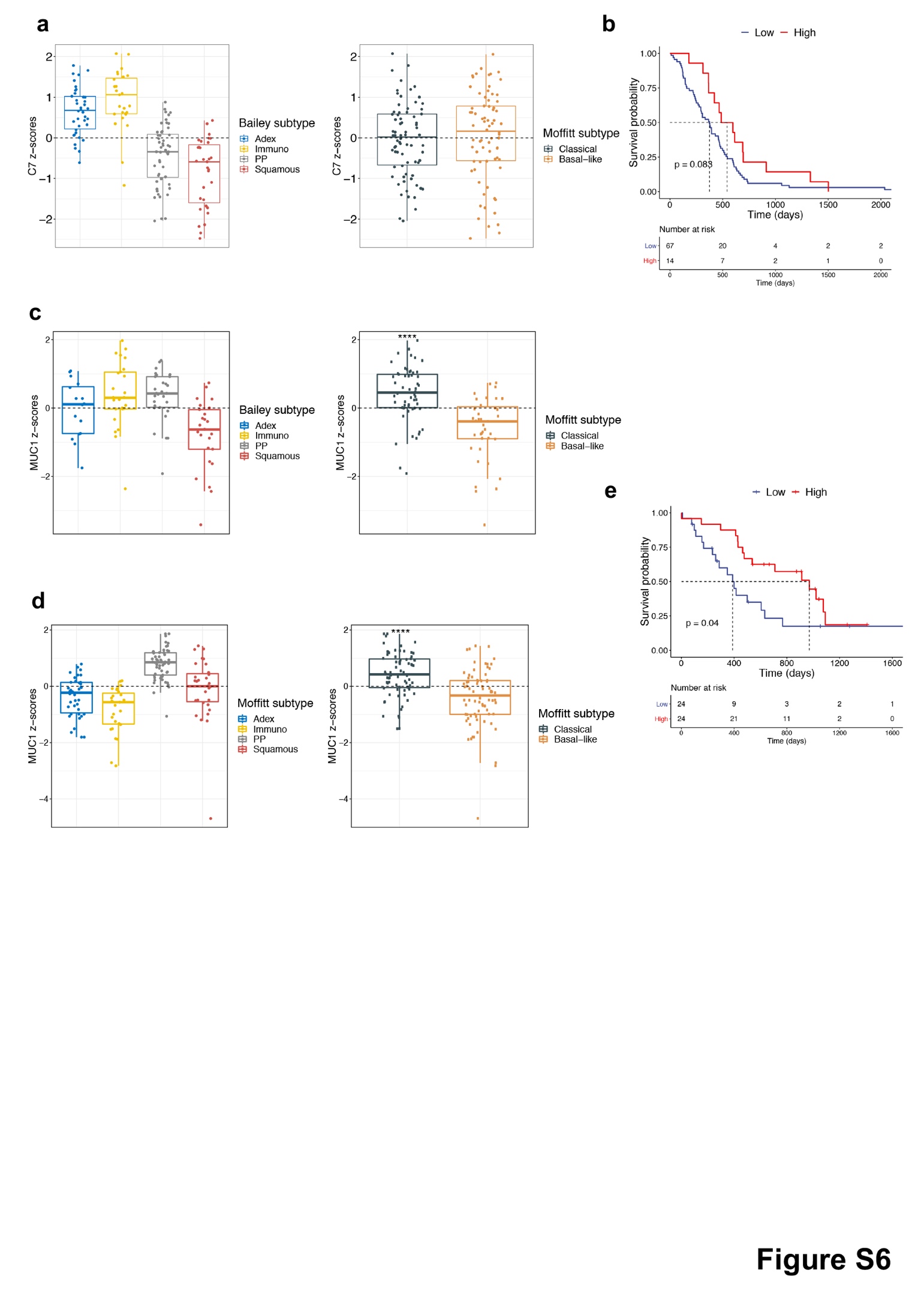


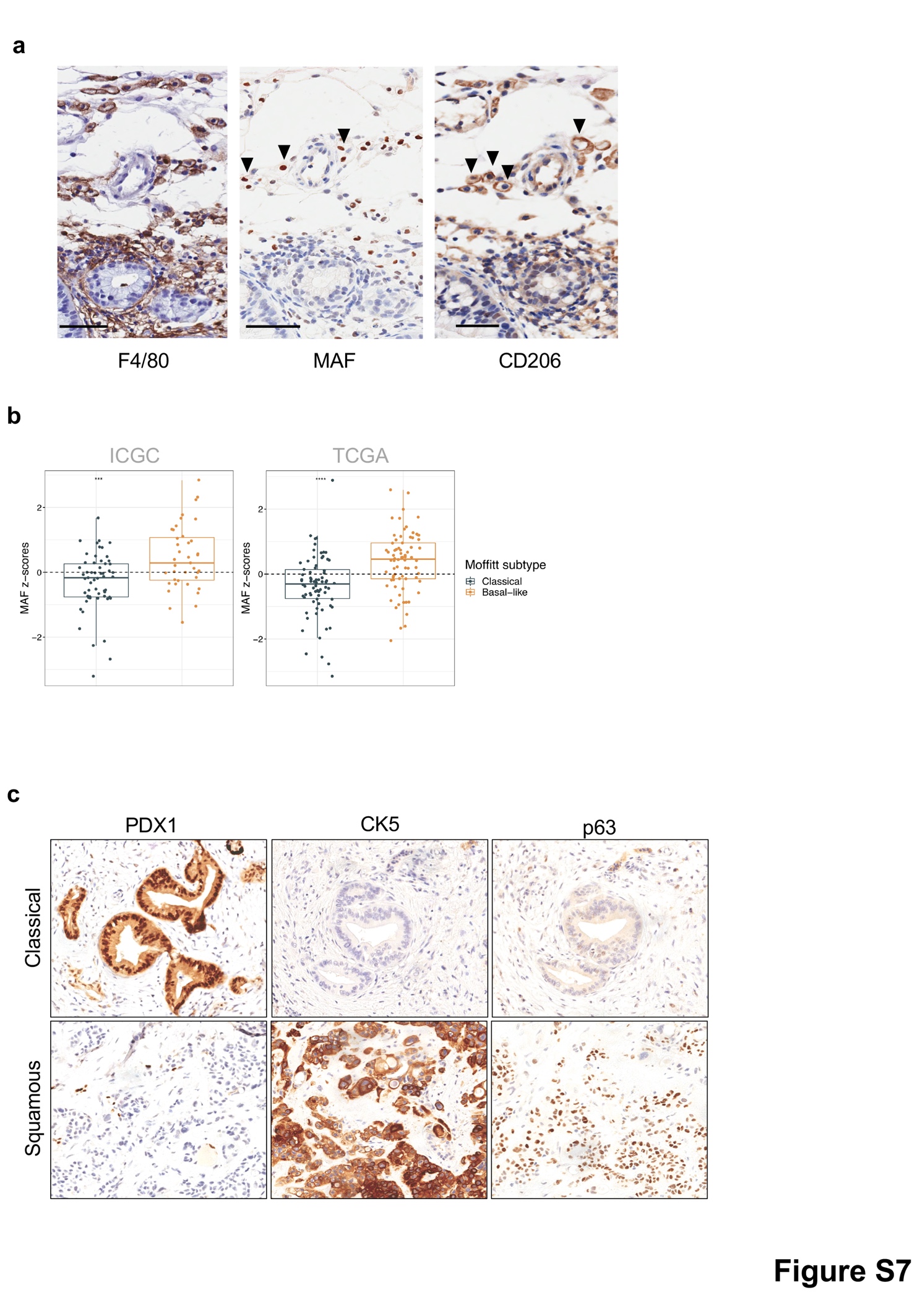

Supplement: Supplementary file 1 — Supplementary Figures [file 41598_2019_48663_MOESM1_ESM.docx]
